# Neural correlates of local parallelism during naturalistic vision

**DOI:** 10.1371/journal.pone.0260266

**Published:** 2022-01-21

**Authors:** John Wilder, Morteza Rezanejad, Sven Dickinson, Kaleem Siddiqi, Allan Jepson, Dirk B. Walther

**Affiliations:** 1 University of Toronto, Toronto, Canada; 2 McGill University, Montreal, Canada; 3 Samsung Toronto AI Research Center, Toronto, Canada; 4 Vector Institute, Toronto, Canada; Justus Liebig Universitat Giessen, GERMANY

## Abstract

Human observers can rapidly perceive complex real-world scenes. Grouping visual elements into meaningful units is an integral part of this process. Yet, so far, the neural underpinnings of perceptual grouping have only been studied with simple lab stimuli. We here uncover the neural mechanisms of one important perceptual grouping cue, local parallelism. Using a new, image-computable algorithm for detecting local symmetry in line drawings and photographs, we manipulated the local parallelism content of real-world scenes. We decoded scene categories from patterns of brain activity obtained via functional magnetic resonance imaging (fMRI) in 38 human observers while they viewed the manipulated scenes. Decoding was significantly more accurate for scenes containing strong local parallelism compared to weak local parallelism in the parahippocampal place area (PPA), indicating a central role of parallelism in scene perception. To investigate the origin of the parallelism signal we performed a model-based fMRI analysis of the public BOLD5000 dataset, looking for voxels whose activation time course matches that of the locally parallel content of the 4916 photographs viewed by the participants in the experiment. We found a strong relationship with average local symmetry in visual areas V1-4, PPA, and retrosplenial cortex (RSC). Notably, the parallelism-related signal peaked first in V4, suggesting V4 as the site for extracting paralleism from the visual input. We conclude that local parallelism is a perceptual grouping cue that influences neuronal activity throughout the visual hierarchy, presumably starting at V4. Parallelism plays a key role in the representation of scene categories in PPA.

## Introduction

Upon opening their eyes, humans experience a rich, cohesive world, composed of many objects and surfaces. We can understand the identity of images presented in a rapid visual stream [1], quickly find objects in noisy real-world environments [2], and even detect shapes composed of smaller elements such as edge segments or Gabor patches [3, 4]. Although it feels effortless, not requiring any thought or concentration by the observer, several stages of processing must occur between light falling onto the retinal array and our conscious percept of the visual world. We have a good understanding of early feature extraction in the striate cortex [5–7] as well as mechanisms of object and scene perception at higher levels [8–10]. In order to arrive at high-level representations from low-level features, the low-level features need to be organized at an intermediate level. This is often called mid-level vision. Principles for organizing features have been established about 100 years ago as Gestalt grouping principles [11]. These principles have been empirically verified with simple lab stimuli, which are easy to control and interpret (for some examples see [12–14]).

Studying perceptual organization in real-world scenes, however, requires image-computable algorithms for detecting grouping cues in such complex images. Our group has recently developed such algorithms for detecting local symmetry and demonstrated that human scene categorization behavior is sensitive to manipulations in the symmetry content of scenes [15, 16]. The current study capitalizes on these algorithmic innovations in order to investigate the neural underpinnings of local parallelism in the perception of real-world scenes. Symmetry has been extensively studied in human vision in many forms. Commonly, *global* forms of symmetry, such as mirror symmetry, are the topic of these studies [4, 17–22]. Here, we focus on one form of *local* symmetry, specifically local translational symmetry, or parallelism, measured via our algorithm for detecting local ribbon symmetry. It captures the extent to which a pair of contours keep a fixed separation. This is true of straight parallel lines, and also parallel lines that bend and weave around, like a ribbon. While we discuss potential relationships to previous studies of global symmetry, our work is only concerned with local symmetry. Specifically, we address two questions: (i) Where is information about local parallelism extracted from the visual input, and (ii) where and how is this information used to facilitate scene perception? We answer these questions in two fMRI experiments.

We address the first question in our second experiment. We explored which brain regions carry a signature of local parallelism by harnessing the natural variability in a large database of natural scenes. A model-based fMRI analysis shows strong signals for local parallelism in area V4, PPA, and retrosplenial cortex (RSC), but also in primary visual cortex (V1). Interestingly, the signal in V1 peaks later than the signal in V4, PPA, or RSC, suggesting a role of neural feedback in the neural presentation of local parallelism in V1. Importantly, this second experiment shows that our measure of local parallelism, which is measured on contours, captures important information about the spatial relationships within real-world photographs.

To address the second question, we showed participants line drawings of complex scenes manipulated in such a way that local parallelism is either degraded or enhanced. This manipulation selectively affects decoding of scene categories in the parahippocampal place area (PPA)—decoding is significantly more accurate when more parallel contours are present.

Our study clearly shows that signals of local parallelism are present throughout the visual processing hierarchy, presumably originating in area V4. The specific role of local parallelism to enhance scene perception, shown previously by our group in human behavior [15] manifests selectively in PPA, the main area responsible for representing scene categories [23, 24].

## Experiment 1

In this experiment, we investigate the effect of local parallelism on the neural representations of scene categories in several visually active brain regions. To this end, we attempted to decode scene categories from patterns of brain activity elicited while observers viewed line drawings containing only the most or only the least parallel contours, as well as intact drawings containing all contours.

### Methods

#### Stimuli

Stimuli were derived from a set of 475 line drawings of six categories of real-world scenes [15, 25]: *beaches*, *forests*, *mountains*, *cities*, *highways*, and *offices*. The line drawings were generated by artists tracing the important contours from a set of color photographs. The artists were given the instruction: “For every image, please annotate all important and salient lines, including closed loops (e.g., boundary of a monitor) and open lines (e.g., boundaries of a road). Our requirement is that, by looking at the annotated line drawings, a human observer can recognize the scene and salient objects within the image.”

**Fig 1:**
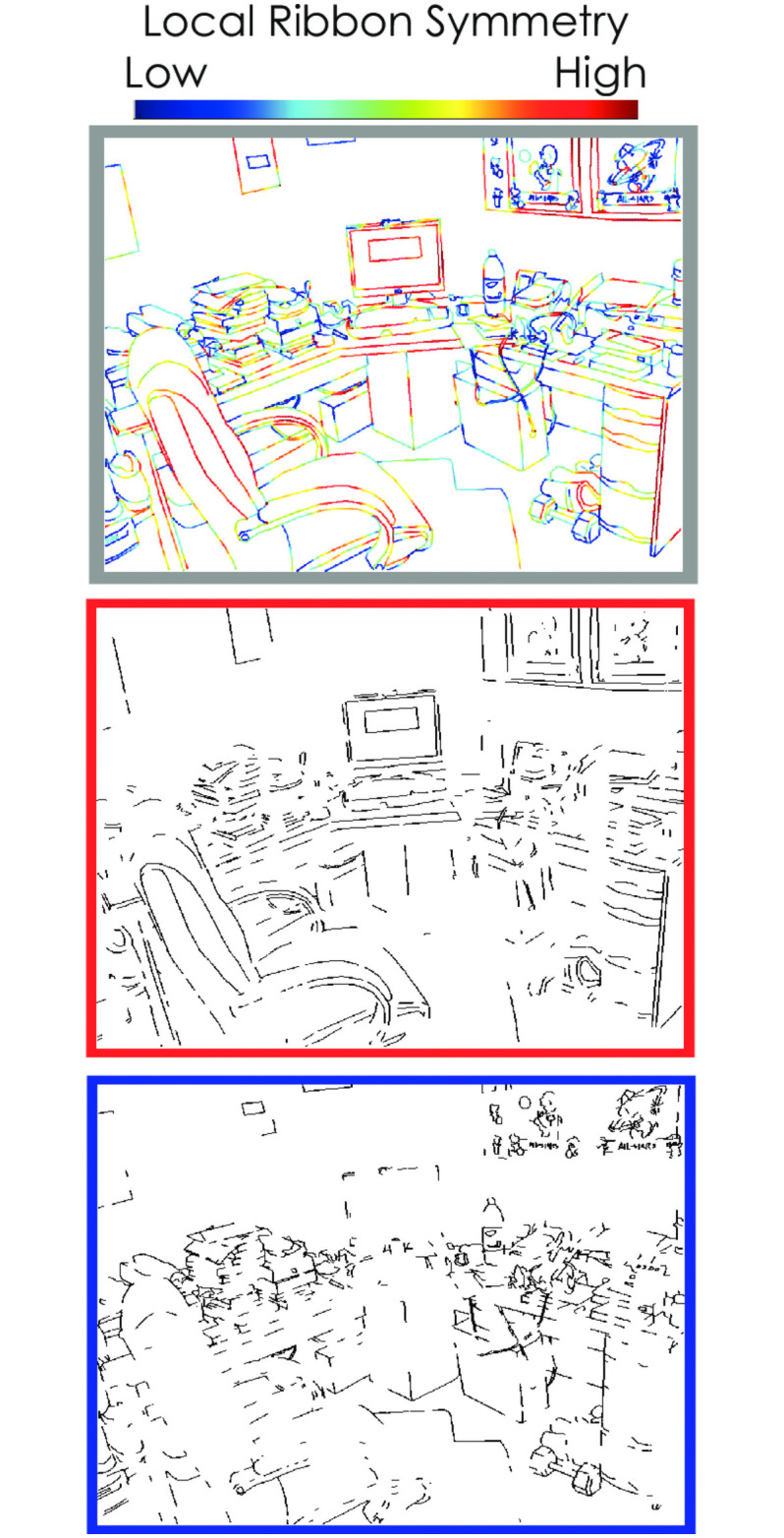
Example office line drawing. The top image (gray border) shows the intact line drawing with contours colored according to their the local ribbon symmetry scores. After the image is scored, the pixels are rank ordered, and the top half is used to create the most parallel image (in red) and the bottom half is used to create the least parallel image (in blue). Each of these images contains exactly half of the contour pixels of the intact image, and combine to form the intact image.

**Fig 2:**
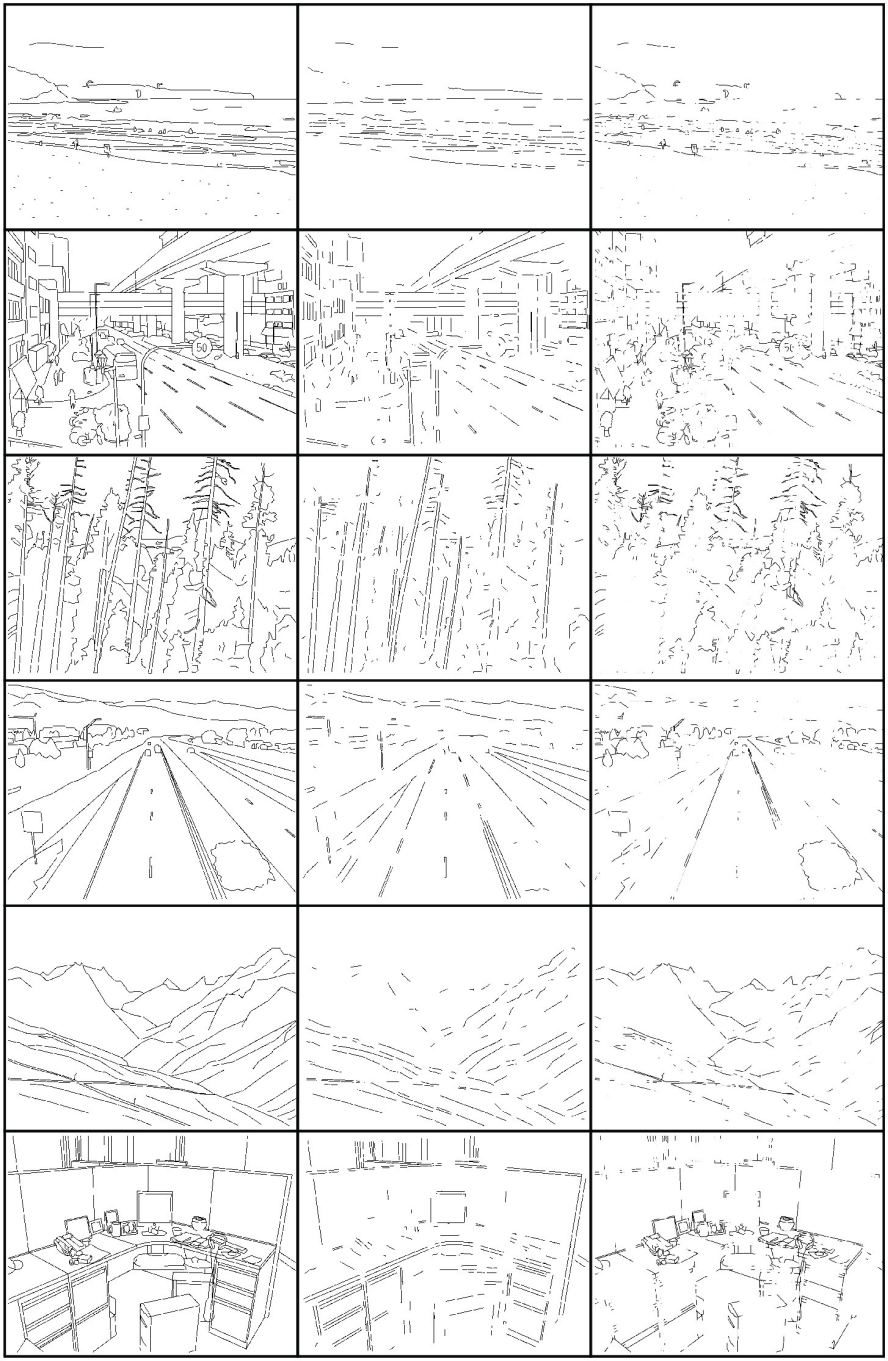
Examples of stimuli from each category (from top to bottom: Beach, City, Forest, Highway, Mountain, Office) and from each condition (from left to right: Intact, most parallel, least parallel).

We scored the individual contours in the drawings according to their local parallelism using an algorithm described in the next section. We created the *most parallel* half-images from the half of the contours with the highest local ribbon symmetry scores and *least parallel* half-images from the half of the contours with the lowest local ribbon symmetry scores. The most parallel and least parallel half-images combine to create the intact image, and they contain an equal number of contour pixels. You can see an example of a scored image, along with the accompanying splits in Fig 1. Additional stimulus examples from each category are found in Fig 2.

In the experiment, we used three versions of each image: intact line drawing, most parallel half-image, and least parallel half image, resulting in 1425 images in total that may be shown to participants.

#### Scoring parallelism via local ribbon symmetry

We measured local parallelism via an algorithm for measuring different types of local symmetry. In this case it is based on local ribbon symmetry. Our algorithm uses the medial axis transform, which allows us to represent a set of contours by the axes between them about which they are locally symmetric. The medial axis consists of the points that are equidistant from two contours points, and is also known as the shape skeleton [26–29].

To determine the medial axis, we first compute the Euclidean distance of each pixel in the image to the nearest contour. The medial axis lies at the points where the gradient of this distance function flows outward (i.e. is multi-valued). We call the distance of the medial axis points to the nearest contour on either side of the medial axis the radius function. We measure the change of the radius function along the medial axis. If there is no change in the radius, the contours on either side of the medial axis are parallel, indicating a locally ribbon-symmetric region. We assign a ribbon symmetry score based on the number of changes in the radius function between neighboring points within a local piece of the medial axis. A small number of changes results in a high symmetry score, and a large number in a low symmetry score.

Once the medial axis is scored, we find the contour points that each axis point flows to (using the gradient of the distance transform). Those contour points receive the scores of their corresponding axis points. As our method finds the medial axis in all white-space regions of an image, there are two shape skeletons that correspond to each contour point, one on either side. We assigned the maximum of the two axis scores to the contour point. More details of this method can be found in [15].

#### Participants

Forty-two participants (26 female, 16 male, mean age 25 years old, age range 18–51 years of age) were recruited from the University of Toronto, from Facebook groups, and from Honeybee (a local service for recruiting study participants), and were paid CAD 40 for their participation. Only 38 participants’ data were used because three participants fell asleep during multiple runs of the experiment and another participant failed the one-back task used to measure if participants were attending to the stimuli.

All participants reported normal or corrected-to-normal vision and provided written informed consent. The experiment was approved by the Research Ethics Board of the University of Toronto (protocol number 33720) and followed the guidelines set out in the Declaration of Helsinki.

#### Design and procedure

We used a block-design fMRI experiment. Participants were scanned in nine experiment runs. Each run contained 18 blocks, one block for each combination of scene category and image condition. In each block, participants were shown 8 scenes from the same category and image condition. Each image was shown for 800 ms, with a 200 ms gap between images. There was a 10 s fixation period prior to the first block and after each block. Participants were instructed to maintain fixation on a centrally presented fixation mark for the entire run. They performed a one-back task, pressing a button on a keypad if they detected that the same scene was shown twice in a row.

Participants underwent functional localizer scans in a separate session. They saw two runs of alternating stimulation of the vertical and horizontal meridians in order to map the boundaries between V1, V2, V3, and V4. In addition, participants saw two runs of a face-place-object localizer, consisting of blocks of images depicting scenes, faces, objects, and scrambled objects. We determined LOC using the contrast between objects and scrambled objects (*q* < 0.001; corrected for multiple comparisons using false discovery rate) [30]. PPA, RSC, and OPA were localized by identifying contiguous clusters of voxels that showed a significant (*q* < 0.001) contrast for (scenes > objects, faces) [31, 32].

#### fMRI data acquisition

Brain imaging data were recorded on a 3 Tesla Siemens Prisma MRI scanner with a 32-channel head coil at the Toronto Neuroimaging Facility (ToNI) at the University of Toronto. High resolution anatomical images were acquired with an MPRAGE (magnetization-prepared rapid acquisition with gradient echo) protocol with multiband factor of 2. Images were then reconstructed using GRAPPA, with sagittal slices covering the entire brain; inversion time (TI) = 912 ms, repetition time (TR) = 1900 ms, echo time (TE) = 2.67 ms, flip angle = 9°, voxel size = 1 x 1 x 1 mm, matrix size = 224 x 256 x 160 mm. Functional images for the retinotopic localizer were recorded with a multiband acquisition sequence; volume repetition time (TR) of 2 s, an echo time (TE) of 35 ms, flip angle of 65 degrees, multiband factor of 4, voxel size of 1.33 x 1.33 x 1.3 mm, matrix size of 200 x 200 x 70 mm, 54 slices at an oblique angle approximately parallel to the calcarine fissure. Functional images for the main experiment and the face-place-object localizer experiment were recorded with a multiband acquisition sequence; volume repetition time (TR) of 2 s, an echo time (TE) of 32 ms, flip angle of 70 degrees, multiband factor of 4, voxel size of 2 x 2 x 2 mm, matrix size of 220 x 220 x 136 mm, 68 axial slices.

#### fMRI data analysis

Data was pre-processed using *fMRIprep* 1.2.6, which is based on *Nipype* 1.1.7 [33]. We used *fMRIprep* in order to have a standardized and repeatable processing pipeline. We kept all parameters at their default settings. Specifically, the T1-weighted images were corrected for intensity non-uniformity [34], skull-stripping, 3D surface reconstruction [35], and segmented into brain tissues [36]. For each functional run a reference volume and skull-stripped version were generated by *fMRIprep*. The volume was then registered to the T1w image [37]. Head motion was estimated and corrected with respect to the reference volume, and the data was resampled in the original space to correct for distortions caused by head motion [38]. Next, the volumes were spatially smoothed (2mm FWHM) and converted to percent signal change using AFNI [39]. Then, also in AFNI, we regressed out motion correction parameters as nuisance variables. We averaged the residuals of this regression over experiment blocks with a hemodynamic delay of 4 s.

We then used a multivariate decoding analysis with the goal of predicting scene categories from the activity patterns of voxels elicited by participants viewing the line drawings [24, 40]. For each ROI, we trained a linear support vector machine (SVM) classifier on the voxel patterns of all experimental runs, except for one run, and then used the trained classifier to predict the scene categories seen during the left-out run. This procedure was repeated so that each run was left out once (leave-one-run-out cross validation), resulting in predicted category labels for all runs. Within ROIs, voxels were selected for inclusion in the analysis based on the rank of the F statistic of a one-way ANOVA of the voxel activity with respect to category label. We set the cutoff for the proportion of voxels to include in the MVPA analysis to the value that gave the highest classification accuracy by the SVM classifier. Voxel selection was performed in a nested cross validation, using only the training data of each cross validation fold. The average number of voxels included based upon the voxel selection ranges from 51 percent of the voxels (for V2) to 59 percent of the voxels (OPA). For individual participants, the proportion of included voxels ranged from including 30 percent to 100 percent.

The SVMs were trained in Matlab using LIBSVM within the CoSMo MVPA toolbox [41]. Separate classifiers were trained for each image condition and tested on data from the same condition. We tested for differences in decoding accuracy between conditions using paired t-tests at the group level, separately for each ROI. We used paired t-tests for these comparisons since we have ad-hoc reasons to suspect a difference between these conditions, see [15]. The decoding accuracy for each subject and ROI is available at: https://osf.io/2g79d/.

### Results

#### Multivariate analysis

**Fig 3:**
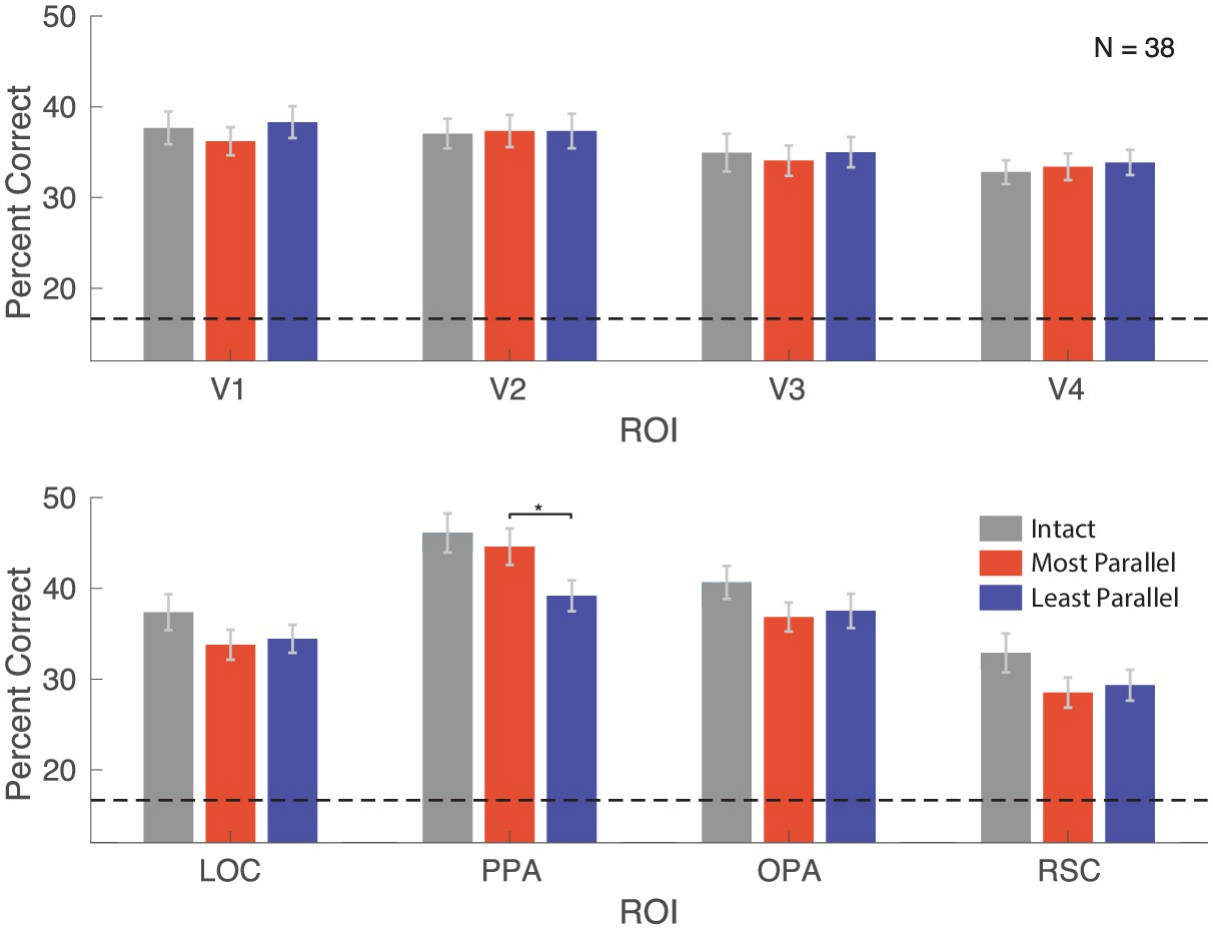
Decoding accuracy for each ROI for intact (gray), most parallel (red), and least parallel (blue) scenes. The dashed line represents chance decoding accuracy. * *p* < 0.01 (FDR-adjusted).

We were able to decode scene categories significantly above chance for all three conditions in all ROIs (Fig 3). We were specifically interested in the relative accuracy between the symmetric and asymmetric conditions. For each ROI, we conducted a paired t-test of decoding accuracy for the symmetric versus the asymmetric condition. We found significantly better performance in the symmetric than the asymmetric condition in PPA (*p* = 0.0018, False Discover Rate “FDR” adjusted *p* = 0.014), but not elsewhere (all *p* > 0.05).

#### Univariate analysis

**Fig 4:**
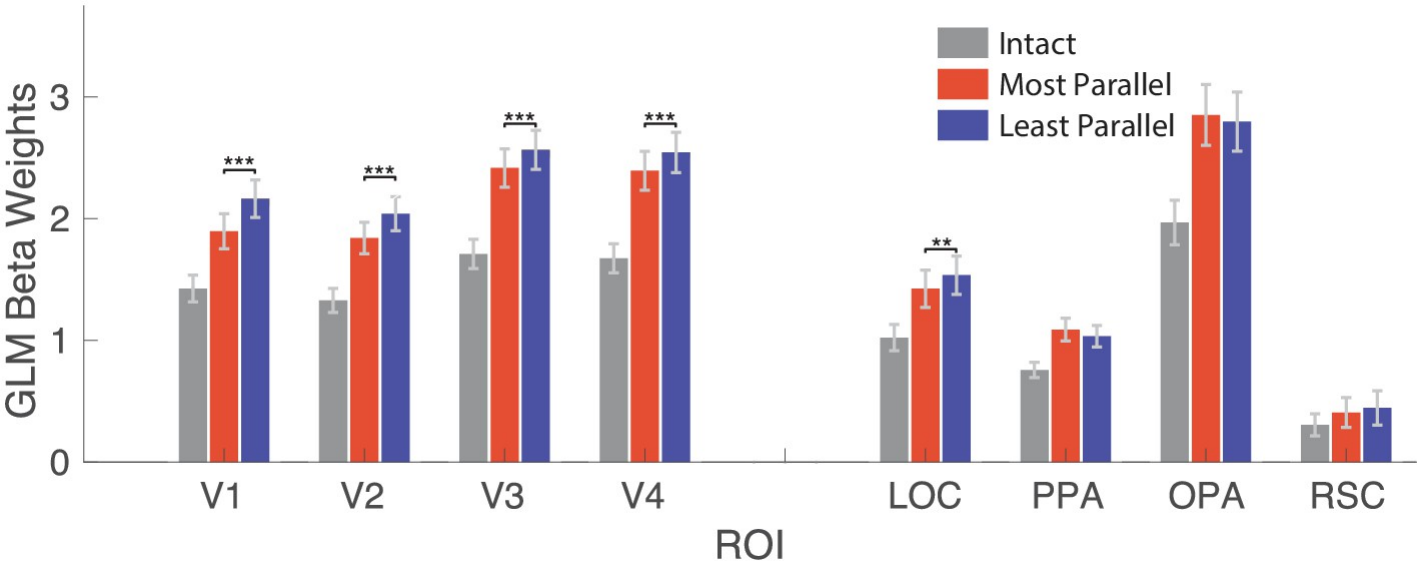
Univariate results. For each ROI, the mean beta weight of the regression analysis is shown (in percent signal change), for intact (gray), most parallel (red), and least parallel (blue) scenes. When using uncorrected p-values, the difference between most parallel and least parallel activity in PPA is significant (*p* = 0.0476). ** *p* < 0.01, *** *p* < 0.001 (FDR-adjusted).

We also analyzed mean BOLD response in each ROI for each image condition (Fig 4). In all ROIs, BOLD activity was lowest for intact line drawings. This comparably low activity level suggests that the intact line drawings are more easily processed, requiring fewer neuronal resources than the two types of degraded line drawings. In V1, V2, V3, V4, and LOC there was significantly more activity in the least parallel condition than the most parallel condition (all FDR adjusted *p* < 0.002), suggesting that in these brain regions, processing of parallel line drawings requires less effort than processing of non-parallel line drawings. Notice that, even though least parallel scenes recruit more activity than the most parallel scenes, this difference is not category-specific, since there was no significant difference in decoding accuracy between the conditions in these brain regions. There were no significant differences (when correcting for multiple comparisons using FDR) in univariate activity in PPA, OPA, and RSC (all FDR adjusted *p* > 0.06, even though PPA exhibited significantly higher accuracy of decoding scene categories from the most parallel than the least parallel half-drawings.

## Experiment 2

In the previous experiment, we carefully controlled the amount of parallelism by splitting line drawings into the most and the least parallel half-images. We found that the representation of scenes in PPA more clearly convey information about scene categories when they contain the most parallel contours, while scene categories are less easily distinguished for scenes containing the least parallel contours. In the second experiment, we explored a neural manifestation of parallelism itself by making use of the natural variation of the amount of parallelism within a large set of scene images. Additionally, we show that while we define local parallelism for a line drawing, it is a more general example of the Gestalt grouping principle that applies also to photographs of real-world scenes. In this experiment, participants saw photographs, and we investigate how the local parallelism measure from line drawings extracted from these photographs is reflected in the time course of BOLD activity in a linear model-based analysis.

### Methods

#### Dataset

For this experiment, we make use of the BOLD5000 dataset [42]. This dataset contains 4916 unique images, taken from ImageNet [43], the COCO dataset [44], and scene images that are inspired, but not directly taken from, the SUN dataset [45]. Four participants were shown the images in a slow, event-related design. They viewed each image for 1 s, followed by a 9 s fixation period. During the fixation period, the participants responded whether they liked or disliked the image. Three participants completed all 15 sessions of functional scans, viewing each image in the dataset. A fourth participant completed only nine functional sessions. All participants completed an additional session, which included a high-resolution anatomical scan. Although the number of participants in this study is smaller than in Experiment 1, the total amount of data is larger. We have a total of 98,940 functional brain images for Experiment 2, corresponding to over 15 hours of scan time for three of the participants and over 9 hours for the fourth. Compare this to 57,114 functional brain images (1,503 for each of 38 participants) in Experiment 1. In fact, extensive scanning of few participants should be preferred over scanning many participant for shorter periods in certain cases [46].

#### Scoring parallelism

**Fig 5:**
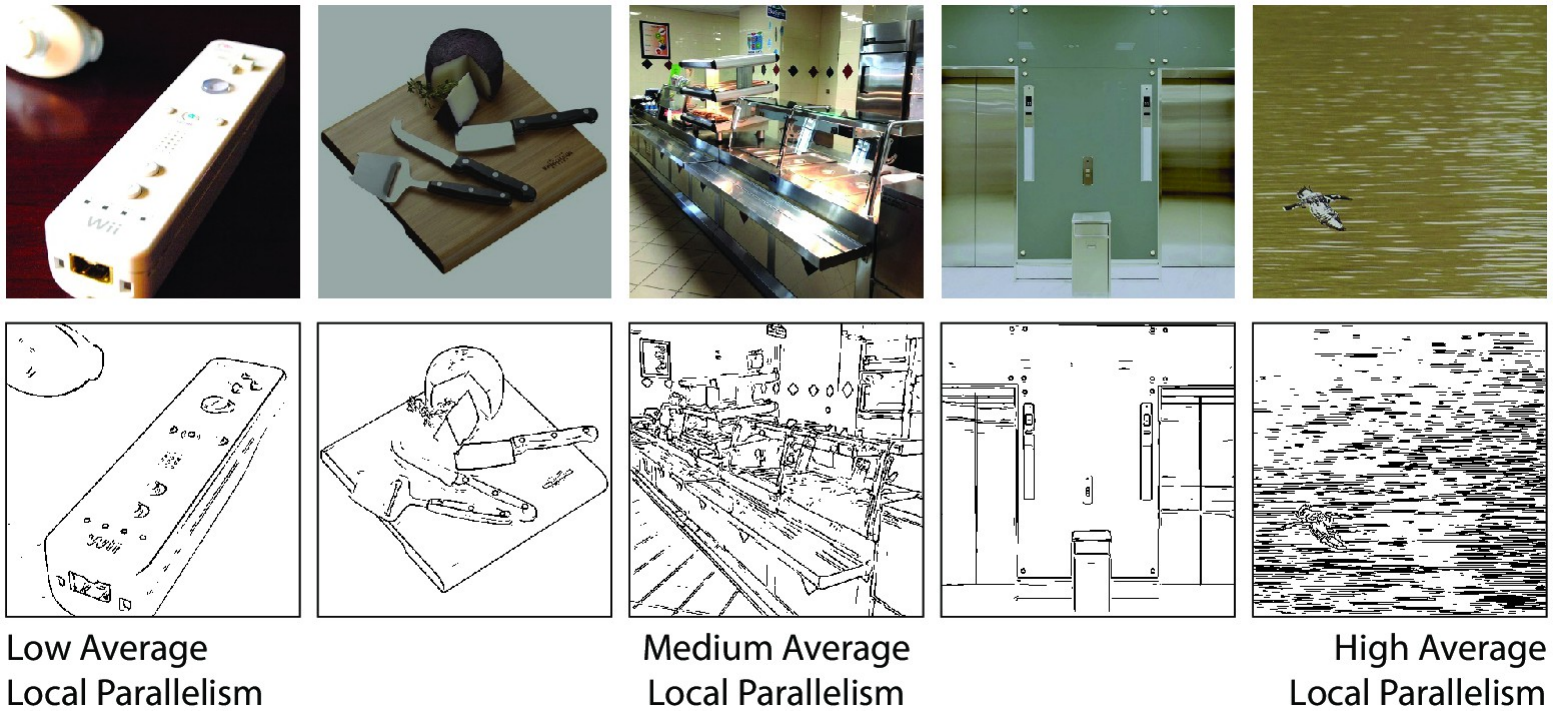
Example photographs used in the collection of the BOLD5000 dataset (top row) and the edges extracted from those images (bottom row). The two leftmost images received some of the lowest average local parallelism, while the two rightmost images had some of the highest average local parallelism. The middle image had an average local parallelism near the middle of the range of the entire dataset.

We converted the color photographs of the BOLD5000 image set into line drawings using an updated version of the logical-linear edge detector [16, 47]; a fast implementation in C is available at: https://github.com/mrezanejad/LineDrawingExtraction. Unlike many other edge detection algorithms, this detector allows for more than one orientation to be present in a given image location, thereby preserving sharp junctions and corners. The contour pixels of the resulting edges were scored for local ribbon symmetry as described in Experiment 1. We then averaged the local ribbon symmetry score over all contour pixels to obtain an overall rating of local parallelism for each image. Example images used to collect the BOLD5000 dataset and the extracted line drawings are shown in Fig 5.

#### fMRI analysis

The fMRI data from BOLD5000 were pre-processed using fMRI-Prep. Pre-processing included brain tissue segmentation, 3D surface reconstruction, obtaining a brain mask/skull stripping, head motion estimation, slice-time correction, and EPI to T1w registration [33]. To assess the role of local symmetry within each image in driving BOLD activity, we constructed a regressor from the average symmetry scores of the images seen by a particular participant. The numbers were aligned with the time of the presentation of the image. We included similarly constructed regressors for mean luminance and mean contrast of the images as covariates. We used AFNI’s 3dDeconvolve function to regress these regressors onto the time course of the BOLD activity for each voxel. We computed the beta-weights from stimulus onset to 12s after image onset using a 7-parameter tent function, thus obtaining one beta weight for each voxel at each acquisition time (TR = 2s).

Our analysis of the symmetry-related beta weights began with an exploratory analysis, where we found the average beta-weight at each stimulus onset and for the 6 TRs post-onset. For each ROI (see how the ROIs were defined below) we fitted a 4-degree polynomial to the beta-weights over time (see Fig 7). This appeared to show that the peak of parallelism related activity for PPA and V4 was prior to the peak of parallelism related activity in V1. To investigate this more closely we analyzed the parallelism related beta weights individually for each observer in a bootrstrap analysis. For each ROI we randomly sampled 50 percent of the voxels from the ROI. We fit a gamma function to the beta-weights over time. For V1, V4, and PPA we determined the mode of the gamma distribution. We recorded if the modes for V4 and PPA were earlier than the mode for V1. We repeated this procedure 10,000 times with a new sample of voxels.

The previously described analysis sampled the same *proportion* of voxels from each ROI. The ROIs have different sizes, and to ensure the size of the ROI did not affect our results we performed a similar analysis but now sampling an equal number of voxels from each ROI. The number of voxels was chosen to be equal to half of the size of the smallest ROI (V4), or 828 voxels. We sampled 100,000 times for each ROI and compared the mode of corresponding samples from each ROI, using the same procedure described in the previous paragraph.

Low-level (V1-V4) regions of interest (ROIs) for further analysis and visualization were determined using a probabilistic map of the visual cortex [48]. V4 was defined using the hV4 map. The probabilistic map gives the likelihood for voxels to belong to each ROI. We assigned voxels to ROIs based on maximum likelihood. For high-level regions of interest we use the atlas of [49]. [49] used data from 30 participants to functionally localize high-level ROIs using the following contrasts: faces vs objects, bodies vs objects, scenes vs objects, and objects vs scrambled objects. They transformed each of their subjects’ data to MNI space and computed the overlap between all participants. The overlap maps were thresholded so only voxels where at least 10% of the participants overlapped. Clusters of voxels were excluded if fewer than 60% of the participants had significant activations within the cluster. Finally, if a cluster matched a location that was previously defined as a category-specific ROI in the literature, it was considered to be part of that ROI. We defined all ROIs bilaterally. After determining the ROI for each voxel in MNI space, we transformed the ROI masks back to the subject space for each participant, as all analyses were performed in the original participant space. We included in our analysis visual areas V1-4, posterior lateral occipital cortex (LO), posterior fusiform sulcus (pFs), parahippocampal place area (PPA), retrosplenial cortex (RSC), and occipital place area (OPA). Another ROI, called *Other*, which contains all voxels that are not in one of the other ROIs, is used as a baseline. The average number of voxels in the ROIs are: V1 *n* = 3968, V2 *n* = 4889, V3 *n* = 4534, V4 *n* = 1656, LO *n* = 16031, pFs *n* = 8659, PPA *n* = 6652, OPA *n* = 5492, RSC *n* = 5291, FFA *n* = 5319, and Other *n* = 134220.

### Results

**Fig 6:**
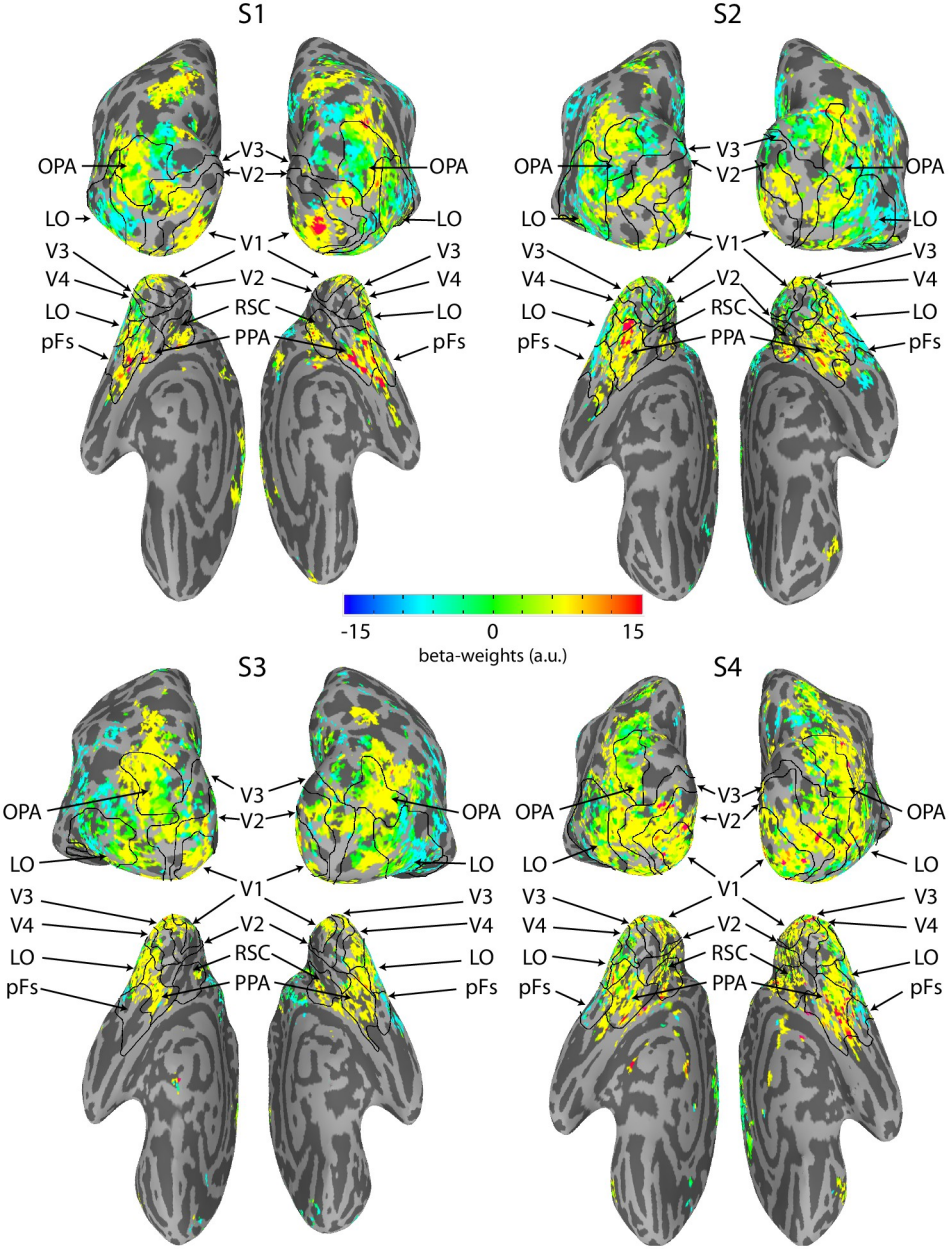
Local parallelism-related brain activity for all participants from the BOLD5000 dataset. The voxels whose activity was significantly influenced by local parallelism are shown in color; cool colors represent negative regression beta weights, and warm colors represent positive beta weights. There is activity throughout the visual pathway. The largest positive values occur in areas V1, V2, and PPA, the most negative weights in area LO.

The regression analysis reveals which voxels are significantly related to the local parallelism above and beyond any influence of the lower-level features (contrast and mean luminance). Fig 6 highlights these voxels. Relevant visual ROIs are marked in black. While there are voxels with activity that is related to local parallelism in all marked visual ROIs, there are fewer such voxels in earlier areas. At higher-level visual areas, especially scene processing areas, such as PPA and OPA, there are many voxels related to parallelism.

**Fig 7:**
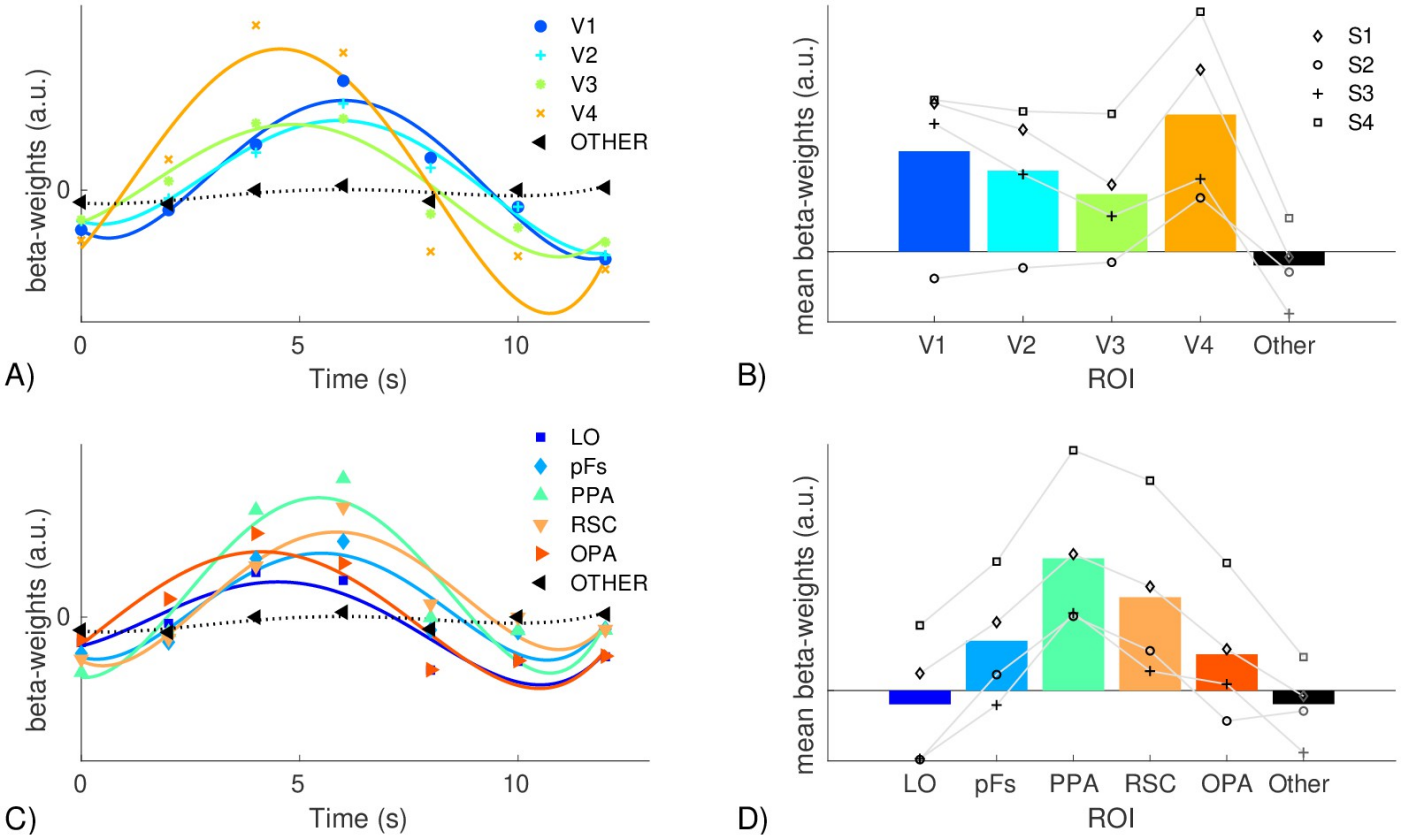
Mean beta-weights showing the strength of BOLD activity influenced by the average local parallelism of a scene. Panels A and C show the beta-weights over time averaged across observers (data points) along with the best fitting 4th-degree polynomial for illustration. Panels B and D show the area under the curves from panels A and C between 2s and 10s, the black markers show the data for each observer. Each observer’s data is connected with a grey line. Panels A and B show the results from early visual areas, whereas panels C and D show the results from later areas. The black curves and bars show the average beta-weights from all voxels in the brain that are not included in any of the other ROIs as a baseline.

In Fig 7 we show the time course of BOLD activity (beta-weights from the GLM) related to local parallelism in each ROI, as well as the mean beta-weight over the time course. Local parallelism-related activity is largest in V4 and PPA, with a large amount of activity in V1 and RSC as well. Local parallelism, by nature, involves the relationship between edges that are spatially separated. Since the receptive fields of V1 neurons are fairly small, they would likely not be be appropriate for detecting local parallelism. This may be why the peak of local parallelism-related activity is later for V1 than either V4 or PPA, as representing such a relationship between contour elements in V1 would require feedback from higher level areas with larger receptive fields.

The bootstrapping analysis using an equal proportion of voxels from each ROI showed that the peak of V4 preceded the peak of V1 in more than 99.6 percent of our samples for each observer. For PPA the peak activity was earlier than V1 more than 99.5 percent of the time for three of the observers. For the fourth observer, PPA had an earlier peak 53.7 percent of the time. Note that this observer also had less data than the other three observers. The bootstrapping analysis where the number of voxels was equated shows the same qualitative results. Most importantly, the peak of V4 preceded the peak of V1 for all samples for three of the four observers, and for the remaining observer, there were only 10 out of 100,000 samples where the peak of V4 did not precede the peak of V1. The PPA versus V1 timing results are also qualitatively the same, but actually slightly stronger for this analysis. For the first three observers we see that the PPA peak significantly preceded the peak for V1 (in 100% of the bootstrapped trials for observer 1, in 99.92 percent of trials for observer 2, and in 99.1 percent of the trials for observer 3). For the fourth observer, the one with less fMRI data than the others, showed the same trend, but the peak of PPA preceded V1 on 91.16 percent of the trials. These analyses suggest that this effect is robust across observers. As the regression analysis included mean luminance and mean contrast as covariates, early activation of V1 following stimulus onset is likely captured by those regressors. This does not suggest that activation in V1 overall occurs later than in other, higher-level areas, but rather the activation related to *local parallelism* occurs later than in area V4 or the PPA. Note that the analysis of Experiment 2 is exploratory in nature, rather than testing a specific hypothesis. Since the dataset contained data from only four participants, we performed the regression for each observer, and chose to display individual results in Fig 6. For Fig 7 we show the individual data points in the right two panes, and the left side shows data averaged across the observers, however the timing analyses were done separately for each observer.

## Discussion

In our two experiments, the amount of local parallelism in an image affects the encoding of the image content in the PPA: More parallel contours result in more easily decodable representations of scene categories in PPA, less parallel contours result in lower decoding accuracy. Furthermore, we have shown that the amount of local parallelism in a large set of color photographs is reflected in the time course of BOLD activity in areas V1, V2, V4, PPA, and RSC.

The univariate results of Experiment 1 show significantly more BOLD activity for the split scenes than for intact scenes, in all ROIs. In areas V1, V2, V3, and LOC there was significantly more activity for the least parallel scenes than the most parallel scenes. The split scenes, by design, have fewer contour pixels than the intact scenes, so the increased activity is not due to having more content in the image. Instead, the visual system presumably recruits more processing resources to interpret the split scenes than to interpret the intact line drawings. This effect is even larger for the least parallel scenes. The multivariate results demonstrate a benefit of parallelism in PPA through more accurate decoding. The earlier areas (V1-V3 and LOC) do not show this benefit. However, a benefit of parallelism can be seen in these areas, as the visual system needs to recruit fewer resources to process the most parallel scenes than the least parallel scenes.

These results suggest that parallelism is important for the early visual system, where parallelism appears to facilitate the process of grouping image content into meaningful units. The early visual areas thus need to do less work when processing the visual input. In area PPA, parallelism is closely tied to the representation of scene categories. When parallelsim is present in a scene, the neural representations of scenes can be more easily discriminated into their natural categories. This highlights the importance of local parallelism in visual processing, which has previously been shown in human behavior [15, 50, 51] and in artificial vision systems [16, 52].

Taken together, our results suggest that there is an effect of parallelism that makes it easier to process a scene in early visual areas, and that a benefit of parallelism persists into higher-level areas, such as PPA, where we see a clearer representation of the most parallel than the least parallel scenes. We believe that these observations are an indication that parallelism is involved in grouping at early stages and once grouped, the visual representations are more useful for visual tasks such as categorization.

The rapid speed at which real-world scenes are processed by the visual system led to the hypothesis that extensive perceptual grouping might not be necessary [53–55]. Instead, smaller elements could be represented as a global collection of features, which could distinguish the different classes of real-world scenes. Rapid scene classification has been argued to rely on many different image properties, such as color, texture, and spatial frequencies [56–59]. However, human observers can rapidly classify *line drawings* of real-world scenes, even though they do not contain the richness of a photograph [25, 60]. This observation suggests that while features like color and texture might be useful to the visual system as it categorizes real world scenes, they are not a necessary part of the categorization process, and perhaps structural features extracted from line drawings may be the key features in scene classification [61]. We have previously shown that behaviorally, relationships between these features are important for scene understanding [15, 62], suggesting that mid-level grouping does occur in rapid scene understanding. These mid-level features function as cues to the geometry of scenes, such as concavity [63] or scene boundaries [9]. The current work shows where this mid-level grouping occurs: V4, PPA, RSC, and even V1 (most likely via feedback).

In partial agreement with our findings, previous work on global mirror symmetry and axiality of shapes (related to the local ribbon symmetry we measure here in order to characterize parallelism) has found an influence of symmetry in V3, V4, and LOC [17, 20, 21, 64–66], with some work suggesting that the main axes of symmetry are processed as early as V1, possibly as a result of feedback [67]. Recently, global mirror symmetry in full color shapes and scenes has been shown to have similar effects in V4 and LOC as previously found with dot patterns [68]. Studies of symmetry using EEG have also found an effect of symmetry on posterior electrodes, starting at around 300ms post stimulus onset [19, 69, 70]. Our results are largely consistent with this previous work. However, we wish to emphasize that our work is looking at a different class of mid-level features. Whereas this previous work measures symmetry globally, across the entire image, resulting in a single measure representing the symmetry in an image, local parallelism measured via local ribbon symmetry can vary across the images. There may be parts of the image with a high amount of parallelism, while other parts of the scene have a low amount of local parallelism. Our method for measuring parallelism is more closely related to the symmetry models of [71, 72]. While these models do capture variability in symmetry across an image by applying local filters across the image, they are designed to represent the mirror symmetry of the scene. The method we use in this paper measures local ribbon symmetry.

The filters used in these previous models are sensitive to texture, whereas our method was designed to capture the local parallelism present in contours of outlines [15]. Our method has been successfully applied to natural images, following the detection of edges, as shown in Experiment 2 and in work spanning computer vision [16] and psychophysics [15, 73]. [74] measured mirror symmetry in closed contours. Their model is a comprehensive shape perception model, looking not only at symmetry, but also other shape properties, such as curvature and orientation. A key difference from our work is that they use an object-centered coordinate frame to characterize the symmetry present in a single object, whereas we are measuring local ribbon symmetry across the entire scene. The local symmetry measured here may most closely relate to the medial axes in [65], so it is not surprising that we find that V4 is activated by stimuli with strong local parallelism. Additionally, we find a large effect in PPA, which has not been previously implicated in representing parallelsim or symmetry. The involvement of the PPA is most likely due to our use of scenes as stimuli. PPA has been shown to have activity consistent with behavioral scene categorization as well as 3D scene geometry [9, 10, 24, 75]. Local parallelism may be related to both of these, which may explain why PPA is affected strongly by parallelism in both of our experiments.

Our results are broadly consistent with the previous work on global mirror symmetry, predominantly studied through the use of dot patterns. Our data, however, would not necessarily be predicted by theories that emphasize the importance of global mirror symmetry. Global mirror symmetry is often argued as important because of its ubiquity in nature [67]. We wish to note, that in the image plane, this is not the case. Generally, a symmetric object will only project to a symmetric image if the camera is at the correct location. Local symmetry, including local translational symmetry or parallelism is robust to changes in camera location [16]. Furthermore, biological forms are often composed of articulating, locally symmetric parts [76–78]. A particular pose of these objects may project onto the retina in a way that results in a globally symmetric image, this is arguably an accidental configuration. Each symmetric part will project to form a local symmetry on the retina. Such local symmetries, representing the projections of 3-D symmetric parts are articulation- and pose-invariant, and thus local symmetry and parallelism may be a more prevalent type of symmetry in our visual world.

The difference between global symmetry and local symmetry may suggest a reason for an apparent contradiction with previous studies on global symmetry. This previous research has suggested that stronger global symmetry results in a higher activation in extrastriate and lateral occipital areas for dot patterns [17, 18], dot patterns on a slanted plane [79], and naturalistic objects and scenes [68]. In Expt. 1 we found stronger local parallelism to result in reduced overall activity. However, we did find more activity *related to parallelism* in early visual areas and the PPA (Expt. 2) as well as clearer representations of scene categories in the patterns of neural activity in the PPA (Expt. 1). We believe the lower activity in early visual areas in Expt. 1 is predominantly explained due to ease of processing, as explained earlier in the discussion. The early visual areas need to group the set of lines into surfaces and objects. They have a much more difficult time doing this for the least parallel images and need to work harder to complete the grouping. This grouping process proceeds by grouping neighboring elements into a larger whole. Global symmetry, on the other hand, is not looking to group neighboring elements based upon their symmetry. Instead, it is looking at distal elements and grouping them based upon symmetry. This is a potentially expensive operation that might require many more resources, explaining the increase in activation in areas that are known to respond highly to globally symmetric stimuli (V3, V4, and LOC). This idea is consistent with [80], who showed that asymmetric dot patterns were represented using more parts-based representations, which consist of local groupings, than symmetric dot patterns, which are represented holistically, requiring longer-ranged interactions.

While much of the previous work has used globally symmetric dot patterns as stimuli, [81] used abstract wallpaper textured patterns. They found that these wallpaper patterns more highly activated V3 when the patterns contained locally rotationally symmetric regions. Using EEG they were able to measure the time course of activation. They found that V3 and V4 had symmetry related activity prior to LOC, suggesting that the symmetry signal originates in V3 and is not present due to feedback from higher layers. While the class of symmetry studied in [81] and [22] was different from the local symmetry studied here, they are more similar than many of the previous symmetry studies. Thus, we hypothesize that the symmetry activation we found in V4 may flow forward to higher layers, such as PPA, and also feed back to V1.

Additionally, theories of object recognition have suggested that symmetry is critical for processing objects [82, 83] as well as object parts and their spatial relations [76]. In fact, contour junctions, which represent such spatial relations, have been found to be important for scene perception [61, 84], as have been middle segments that represent symmetry-type interactions between parts [62]. Local symmetry was indeed found to be a powerful cue to scene understanding [15], and even to direct eye gaze when viewing scenes [85]. The influence of local symmetry in scene understanding cannot be explained by other simple contour features, such as contour length, orientation, or junctions [15, 62].

Since parallelism is critical for processing objects and object parts, perhaps our most parallel scenes contain more objects. Is our decoding advantage in PPA due to the most parallel scenes containing more objects or closed contours? We counted the number of recognizable objects in the most parallel scenes and least parallel scenes from Expt. 1. The most parallel scenes contained, on average, 0.31 recognizable objects, while least parallel scenes contained 0.84 recognizable objects. Additionally, we computed the number of closed regions in each scene. The most parallel scenes had, on average, 2.61 closed regions, while the least parallel scenes contained 22.02 closed regions. This analysis suggests one possible reason why univariate activite is higher in early visual cortex through LOC. [86] found that V1 and LOC respond more strongly to scenes with more clutter. Having more closed regions would occur in a more cluttered scene, so we see our data as consistent with their results. Relating to our multivariate analysis we think this result suggests that finding recognizable objects is not necessary to classify a scene. While objects tend to be symmetric in the three dimensions, their projections into the image plane are generally not symmetric. We therefore find fewer objects in most parallel scenes but nevertheless the most parallel scenes are easier to decode in PPA.

 [87] showed that PPA may be involved more generally in perceptual grouping. In their study, PPA activated strongly for real-world regularities in images. Local parallelism is just one example of these real-world regularities; we predict that other perceptual grouping cues would similarly drive PPA.

In order to understand the visual processing of the complex visual stimuli encountered in our daily lives, it is useful to have models that are image-computable. The results from our second experiment demonstrate that even though we measured local parallelism in a line drawing, the grouping information extracted is related to the BOLD activity of participants viewing the photos from which those line drawings were extracted. Thus, our measure could be part of a system that takes a photograph as input, extracts contours and carries out perceptual grouping of the contours.

Human scene processing is aided by strong perceptual grouping cues, such as parallelism. Scenes with stronger cues to parallelism more strongly activate visual areas throughout the visual hierarchy, specifically V1, V4, PPA, and RSC. We also show that scene-selective cortex represents the categories of scenes with stronger perceptual grouping cues more clearly in their voxel patterns. Scene representation is thus not only facilitated by the presence of strong grouping cues, allowing the scenes to be processed rapidly, but those grouping cues result in a representation that more clearly encodes important scene information, namely scene categories.

## Supporting information

S1 Appendix (PDF)
