# Supplementary material for: Neural correlates of local parallelism during naturalistic vision
Source: PLoS One. 2022 Jan 21;17(1):e0260266. doi: 10.1371/journal.pone.0260266 (PMC8782314; doi:10.1371/journal.pone.0260266)
Supplement: S1 Appendix — (PDF) [file pone.0260266.s001.pdf]

Additional Decoding Analyses

We have no specific hypotheses about how males and females should or should not differ with respect to any conditions in our study. In vision science there is little data to inform hypotheses about sex-based differences. In order to support researchers who wish to investigate these sorts of differences, in addition to performing the MVPA described above, we separated our dataset into females (n=24) and males (n=14). We performed t-test for each ROI, checking for a difference between the intact performance between males and females. All these t-tests were non-significant (all $p > 0.18$). Additionally, we performed a linear mixed-effects ANOVA looking for a main effect of female vs male and a main effect of most parallel vs least parallel. There was only a main effect of most parallel vs least parallel in PPA, all other $p > 0.37$. OPA showed a main effect of female vs male, all others did not, with $p > 0.38$.

The mean and standard deviation for the percent correct decoding accuracy in each ROI is shown in tables 1 (female) and 2 (male).

Overall, we see similar results in early visual cortex, with decoding accuracies between 30 and 40 percent, and no significant differences between image conditions.

With females (see Fig. 8), we see the difference between decoding scene category from PPA from most parallel versus least parallel areas is slightly larger than in the full

	Intact	Most Parallel	Least Parallel
V1	36.73 (12.13	36.65 (10.27)	36.34 (9.63)
V2	37.65 (10.51)	37.11 (11.72)	35.80 (9.96)
V3	35.26 (13.86)	33.64 (9.61)	33.87 (9.61)
V4	33.27 (9.15)	33.64 (10.30)	34.49 (9.63)
LOC	36.50 (10.47)	34.34 (11.93)	33.49 (8.33)
PPA	46.37 (15.36)	44.44 (13.31)	37.65 (10.57)
OPA	39.35 (12.18)	34.95 (10.10)	34.88 (11.28)
RSC	30.71 (13.12)	26.62 (10.46)	28.63 (11.28)

Table 1. Average decoding accuracy for female participants for each ROI. Standard deviation is in parenthesis.

	Intact	Most Parallel	Least Parallel
V1	39.29 (8.78)	35.45 (7.89)	41.67 (11.77)
V2	35.98 (9.18)	37.70 (9.38)	39.95 (13.81)
V3	34.39 (10.54)	34.78 (11.47)	32.80 (11.15)
V4	32.01 (5.69)	32.94 (6.08)	32.80 (6.31)
LOC	38.89 (14.36)	32.80 (5.88)	36.11 (11.08)
PPA	45.63 (8.43)	44.84 (10.61)	41.80 (10.03)
OPA	42.86 (8.71)	40.08 (8.36)	42.06 (10.76)
RSC	36.64 (12.29)	31.75 (8.65)	30.55 (8.53)

Table 2. Average decoding accuracy for male participants for each ROI. Standard deviation is in parenthesis.

sample. We also find a non-significant effect in the opposite direction in RSC, where decoding is better for least parallel scenes than most parallel scenes.

With males (see Fig. 9), we see a smaller effect in PPA. The effect is no longer significant, but is in the same direction as the full sample. Males have no effect in RSC, and in fact show a non-significant trend in the opposite direction of females. Additionally, males show a non-significant reverse effect in LOC, where decoding of least parallel scenes is easier than most parallel scenes.

We repeat the univariate analyses, again, separating females (Fig. 10) and males (Fig. 11). The early visual area results match that of the larger sample, for both females and males. The least parallel scenes resulted in stronger activity than the parallel scenes. Distinct from the entire sample, males did not show a difference in activity between most parallel and least parallel scenes in LOC. Finally, overall activity in RSC is lower for females than for males.

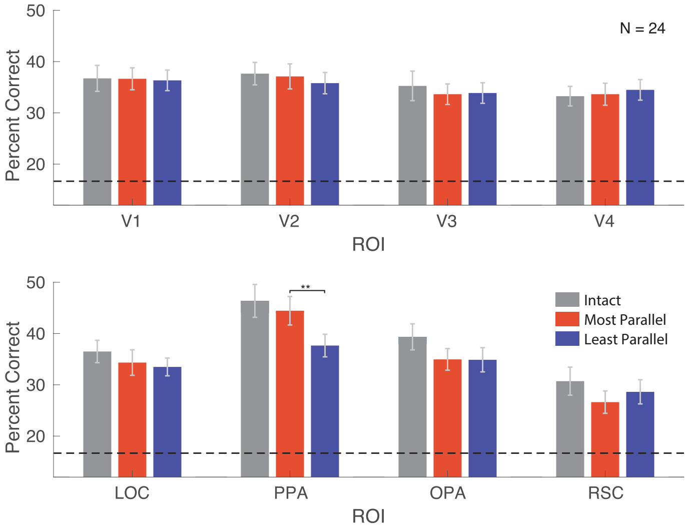

Fig 8. Female participants' decoding accuracy for each ROI (V1, V2, V3, V4, LOC, PPA, OPA, RSC) for intact (gray), most parallel (red), and least parallel (blue) scenes. ** $p < 0.01$ (FDR-adjusted)

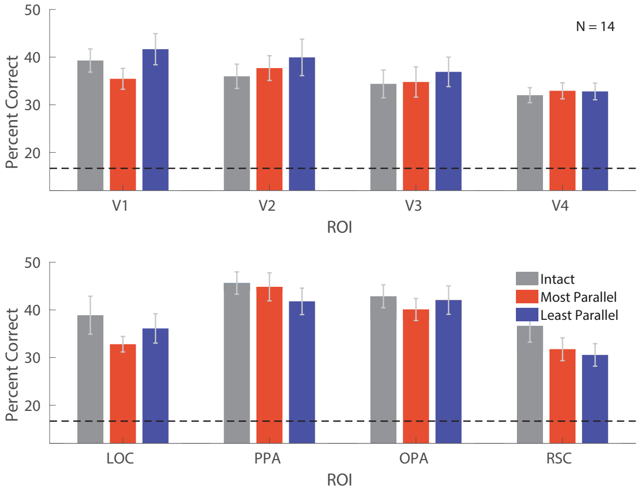

Fig 9. Male participants' decoding accuracy for each ROI (V1, V2, V3, V4, LOC, PPA, OPA, RSC) for intact (gray), most parallel (red), and least parallel (blue) scenes.

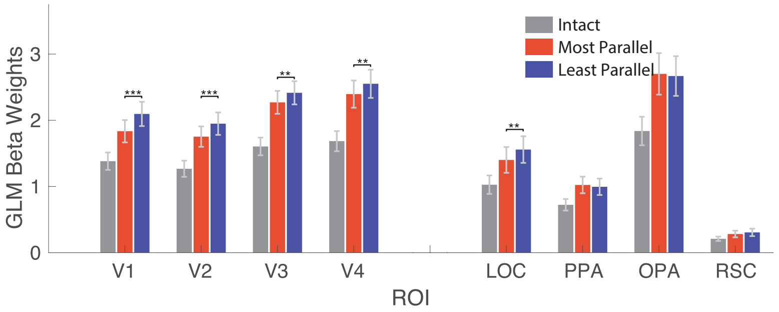

Fig 10. Female participants' univariate analysis. For each ROI, the mean beta weight is shown, for intact (gray), most parallel (red), and least parallel (blue) scenes. ** denotes FDR adjusted $p < 0.01$, and *** $p < 0.001$ (FDR-adjusted).
https://www.overleaf.com/project/60de061b57afd7fd549c6c6e

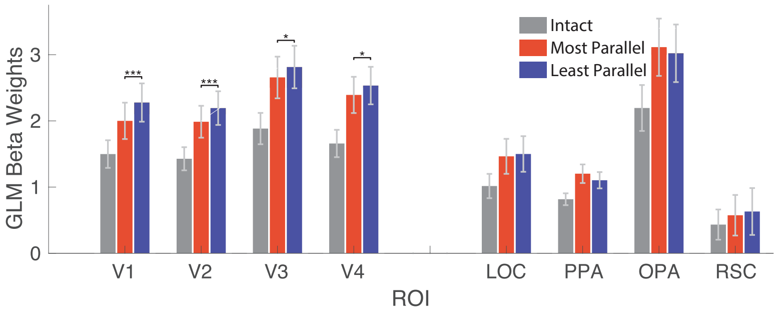

Fig 11. Male participants' univariate analysis. For each ROI, the mean beta weight is shown, for intact (gray), most parallel (red), and least parallel (blue) scenes. * denotes FDR adjusted $p < 0.05$, and *** $p < 0.001$ (FDR-adjusted).
